# Molecular Crossroads: An Expansive Review of Oxytocin’s Impact on SIRT1 Activation and Implications for Health

**DOI:** 10.31661/gmj.v13i.3307

**Published:** 2024-12-31

**Authors:** Yuki Ueda

**Affiliations:** ^1^ UNI H&H Graduate School, Osaka 564-0051, Japan

**Keywords:** AMPK, Metabolic Homeostasis, PI3K, MAPK

## Abstract

Oxytocin, once known for its role in social bonding and reproduction, now reveals multifunctionality with profound effects on health. This paper explores the interplay between oxytocin and SIRT1, a key regulator of cellular health, shedding light on their combined impact on well-being. Oxytocin and SIRT1’s roles in the body are introduced, with the former influencing maternal behavior, social bonding, and emotional regulation, while the latter orchestrates essential cellular processes like DNA repair and metabolism. Through a thorough analysis of existing research, the review delves into the molecular mechanisms through which oxytocin influences SIRT1 activation and expression, fine-tuning cellular functions through downstream signaling cascades. The impact of oxytocin-induced SIRT1 activation on various aspects of health is central to the discussion. From neuroprotection to cardiovascular health and immune modulation, the review highlights the far-reaching implications of this molecular crossroads on overall well-being. Potential therapeutic applications of targeting the oxytocin-SIRT1 axis are also explored, emphasizing its promise in treating diverse health conditions. While valuable insights are gleaned from existing knowledge, the review identifies research gaps and outlines future perspectives. Further investigations are needed to fully understand the interactions between oxytocin and SIRT1, unlocking potential for novel medical interventions. In conclusion, this review establishes the significance of the molecular crossroads formed by oxytocin and SIRT1 in influencing human health. Their intertwining opens new avenues for scientific exploration and therapeutic advancements, holding promise for enhancing human well-being and combating various diseases.

## Introduction

Oxytocin: A Multi-faceted Neuropeptide

Oxytocin, a nonapeptide hormone, was originally recognized for its pivotal role in reproductive functions, particularly during childbirth and lactation, where it facilitates uterine contractions and milk ejection, respectively. However, over the years, research has unraveled a myriad of other functions, positioning oxytocin as a multifunctional neuropeptide with far-reaching effects on various physiological and behavioral processes [1].

One of the most intriguing aspects of oxytocin is its involvement in social bonding and emotional regulation. Studies have shown that oxytocin plays a crucial role in promoting prosocial behaviors, such as trust, empathy, and altruism, which are essential for fostering social connections [2]. Oxytocin has been implicated in enhancing maternal-infant bonding and strengthening the bond between romantic partners [3][4]. Moreover, it has been associated with increased feelings of social closeness and reduced social anxiety [5].

Oxytocin is also involved in the regulation of the stress response and anxiety. Research suggests that oxytocin can attenuate the activity of the hypothalamic-pituitary-adrenal (HPA) axis, the central stress response system, leading to a reduction in cortisol levels and stress-induced behaviors and promote adaptive stress coping strategies and resilience to stress [6][7]. Oxytocin’s anxiolytic properties have been observed in various preclinical and clinical studies, highlighting its potential as a therapeutic target for anxiety-related disorders [8].

In addition to its role in maternal bonding, oxytocin has been implicated in paternal and caregiving behaviors. Studies in both humans and animals suggest that oxytocin promotes parental caregiving behaviors, fostering nurturing and protective actions towards offspring [9]. Moreover, oxytocin administration has been shown to enhance caregiving responses in non-parental individuals as well, highlighting its broader significance in caregiving and affiliative behaviors [10].

Research has demonstrated that oxytocin can modulate social perception and recognition. It enhances the processing of social cues, such as facial expressions, voices, and body language, promoting better social communication and understanding. Moreover, oxytocin appears to influence social memory, facilitating the recognition and recall of familiar individuals [11][12].

## SIRT1: The Master Regulator of Cellular Homeostasis

Sirtuin 1 (SIRT1), a member of the sirtuin family of NAD+-dependent histone deacetylases, has emerged as a critical regulator of cellular homeostasis with profound implications for health and longevity [13]. SIRT1 exerts its influence by orchestrating a diverse array of cellular processes, ranging from gene expression and DNA repair to metabolism and stress response [14]. In this section, we delve into the multifaceted roles of SIRT1 and its impact on various aspects of cellular function and overall well-being.

SIRT1 plays a crucial role in epigenetic regulation by deacetylating histones, which leads to chromatin remodeling and subsequent alterations in gene expression [15]. By deacetylating transcription factors, SIRT1 can influence the expression of genes involved in a wide range of cellular functions, including metabolism, cell cycle regulation, and stress response.

SIRT1 is implicated in maintaining genome integrity and stability. It promotes DNA repair pathways, such as base excision repair and double-strand break repair, thereby protecting cells from genomic damage and reducing the risk of mutations and genomic instability [16].

SIRT1 plays a central role in metabolic regulation, particularly in energy metabolism and glucose homeostasis. It modulates the activity of key metabolic regulators, such as peroxisome proliferator-activated receptor-gamma coactivator 1-alpha (PGC-1α) and AMP-activated protein kinase (AMPK), to enhance mitochondrial biogenesis, increase fatty acid oxidation, and improve insulin sensitivity [17][18][19][20][21].

SIRT1 has garnered significant attention for its potential role in regulating aging processes. It is implicated in the regulation of cellular senescence and lifespan extension in various model organisms [22][23]. Activation of SIRT1 has been shown to promote longevity and delay age-related phenotypes.

SIRT1 has been linked to neuroprotection and cognitive function. It plays a role in neuronal survival and synaptic plasticity, and its activation has been shown to enhance cognitive performance in animal models [24][25].

SIRT1 exerts protective effects on the cardiovascular system by regulating endothelial function, vascular tone, and inflammation. It has been implicated in maintaining vascular integrity and reducing the risk of atherosclerosis [26][27].

SIRT1 has immunomodulatory effects and can influence the inflammatory response. It regulates the activity of nuclear factor-kappa B (NF-κB) and other inflammatory mediators, thereby attenuating the inflammatory cascade and promoting immune homeostasis [28][29].

SIRT1 plays a role in cellular stress response pathways, such as the heat shock response and oxidative stress defense. By deacetylating and activating transcription factors involved in stress adaptation, SIRT1 helps cells cope with various stressors [30][31].

SIRT1 has been implicated in tumor suppression and cancer prevention. It can influence cell cycle progression, DNA repair, and apoptosis, thereby restraining tumorigenesis and promoting cellular health [32][33][34]. It also affects lipid metabolism by regulating the activity of sterol regulatory element-binding proteins (SREBPs), which control the synthesis of cholesterol and fatty acids [35][36].

## Mechanism of How Oxytocin Enhances SIRT1 Activity

The interplay between oxytocin and SIRT1 in cellular signaling has garnered significant interest due to its potential implications for various physiological processes and health outcomes. While the precise mechanism of how oxytocin enhances SIRT1 activity is not yet fully elucidated, emerging research has shed light on several molecular pathways and interactions that may contribute to this phenomenon. This section explores the proposed mechanisms of how oxytocin influences SIRT1 activity and its potential implications for cellular regulation and overall health.

Oxytocin Receptor Signaling and SIRT1 Activation:

Oxytocin signals through its specific G-protein coupled receptor, the oxytocin receptor (OXTR). Activation of OXTR triggers downstream signaling cascades, including the phosphoinositide 3-kinase (PI3K)/protein kinase B (Akt) pathway [37]. Evidence suggests that the PI3K/Akt pathway may be involved in SIRT1 activation. Akt has been shown to directly phosphorylate SIRT1 at specific sites, leading to enhanced SIRT1 activity [38].

Epigenetic Regulation of SIRT1 Expression:

Oxytocin has been implicated in epigenetic modifications that may affect SIRT1 expression. For instance, oxytocin has been shown to modulate DNA methylation patterns in certain gene promoters [39]. DNA methylation changes in the SIRT1 promoter region could alter its transcriptional regulation, influencing SIRT1 expression and activity [40].

Crosstalk with Other Signaling Pathways:

Oxytocin signaling intersects with various other pathways that are known to modulate SIRT1 activity. For example, oxytocin has been reported to activate the mitogen-activated protein kinase (MAPK) pathway, which has been linked to SIRT1 regulation [41][42]. Additionally, oxytocin can influence the AMP-activated protein kinase (AMPK) pathway, a key regulator of cellular energy metabolism, which also interacts with SIRT1 [43].

Modulation of MicroRNAs:

MicroRNAs (miRNAs) are small non-coding RNAs that post-transcriptionally regulate gene expression. Oxytocin has been found to alter the expression of specific miRNAs [44]. These miRNAs could potentially target SIRT1 mRNA, thereby affecting SIRT1 protein levels and activity [45].

Despite the progress made in understanding the mechanisms underlying the oxytocin-SIRT1 interaction, further research is needed to fully characterize this molecular crossroads comprehensively. The multifaceted interplay between oxytocin and SIRT1 likely involves a complex network of signaling events and regulatory elements, contributing to the diverse effects observed in various cellular processes and health outcomes.

## Conclusion

The oxytocin-SIRT1 axis represents a fascinating molecular crossroads with diverse implications for cellular regulation and health. The evidence presented in this review suggests that oxytocin can modulate SIRT1 activity through various molecular pathways, impacting processes such as gene expression, DNA repair, metabolism, and stress response. Harnessing the therapeutic potential of this axis could offer novel strategies for targeting age-related disorders, metabolic diseases, and neurodegenerative conditions. However, further research is warranted to fully elucidate the mechanisms and therapeutic implications of the oxytocin-SIRT1 axis for human health.

## Acknowledgments

The author would like to express sincere gratitude to all individuals and organizations whose support and contributions made this work possible.

## Conflict of Interest

The author declares no conflict of interest.
